# Supplementary material for: Risk of Cancer in Connective Tissue Diseases in Northeastern Italy over 15 Years
Source: J Clin Med. 2022 Jul 22;11(15):4272. doi: 10.3390/jcm11154272 (PMC9332163; doi:10.3390/jcm11154272)
Supplement: Supplementary file 1 [file jcm-11-04272-s001.zip › jcm-1782799-supplementary.pdf]

Supplementary Materials

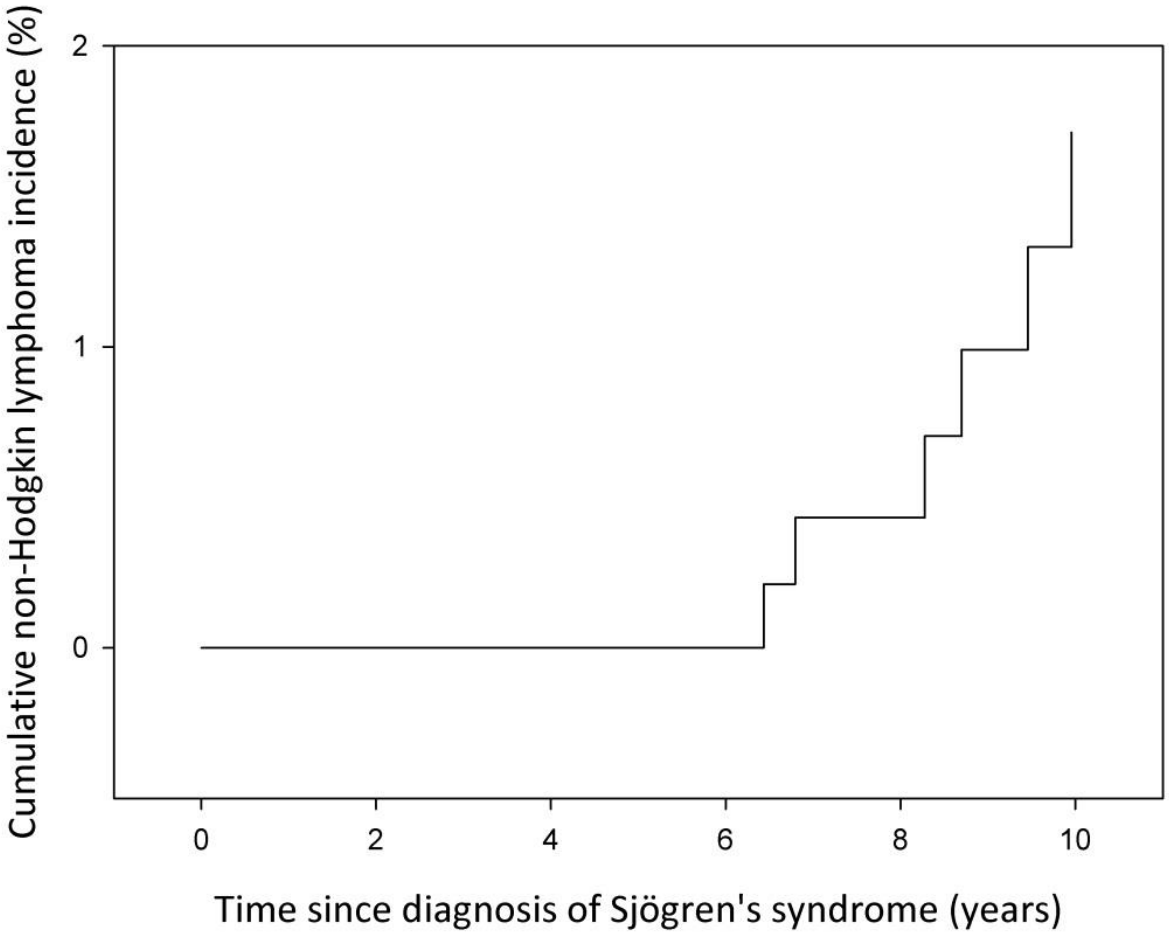

Figure S1. Cumulative NHL incidence by time since diagnosis of Sjögren's syndrome. Friuli Venezia Giulia, 2002-2017. Cancers diagnosed within the first 90 days were excluded.

Table S1. ICD9-CM codes reported in hospital discharges, exemption codes, and ATC codes used for selections and exclusions.

Selections/Exclusions	Conditions	ICD9-CM Codes in Hospital Discharges	Exemption Codes	ATC Codes in Drug Prescriptions
Selections	Systemic lupus erythematosus	710.0	028	
	Systemic sclerosis	710.1	047, RM0120	
	Sjögren's syndrome	710.2	030	
	Dermatomyositis	710.3	RM0010	
	Polymyositis	710.4	RM0020	
Exclusions	Rheumatoid Arthritis	714	006	L04AB04<br>L04AB01<br>L04AB02<br>L04AB05<br>L04AB06<br>L04AC03<br>L04AC05<br>L04AC10<br>L04AC13<br>L04AA32<br>L04AC07<br>L04AA24
	Psoriatic Arthritis	696	045	
	Ankylosing Spondylitis	720	054	

ATC: Anatomical Therapeutic Chemical Classification System.
